# The Institutional Library

**Published:** 1920-09-04

**Authors:** 


### THE INSTITUTIONAL LIBRARY.

**Venereal Diseases: their Clinical Aspect and Treatment.** By J. E. R. McDonagh, F.R.C.S., Surgeon to the London Lock Hospital. (London: Wm. Heinemann. Price £3 3s. net.)

To those who seek a complete and beautifully illustrated treatise on venereal diseases in all their clinical aspects we can heartily recommend Mr. McDonagh's volume of 400 or so pages. There is no aspect of this work which is not more or less exhaustively dealt with in his pages, and although some of the author's theories, particularly in connection with syphilis and its treatment, have not met with universal acceptance, yet their inclusion is fully justified and makes his publication something more than the mere venereal text-book, a class with which the doctor has been inundated during the last year or two. Of the majority of treatments suggested complete case records are given, and although inspection of these does not lead one to think that all the methods are infallible, yet Mr. McDonagh has inserted clinical and historical detail galore, with just that degree of optimism and faith in his own views which provides at once both entertaining and stimulating reading. The publishers themselves are to be heartily congratulated on the general production of the book, which is excellent.

**The Diary of a Police Surgeon.** By Graham Grant, V.D., Lt.-Col. R.A.M.C.T., L.R.C.P.&S. (Edin.), Barrister-at-Law, etc. (London: C. Arthur Pearson, Ltd., Henrietta Street, W.C. 2.)

This unpretentious little volume is made up of sketches of the people whom Col. Grant met during his thirty years' practice in the East-End of London, chiefly in his capacity of police surgeon. The incidents he relates are facts, nor has he tried to dress them up in sentimental fashion. But because of the simplicity of narration they are the more impressive, and although many of the tales are sad enough, Col. Grant never loses his sense of humour. A chapter (or "leaf," as the author prefers to term it) which is of special interest is that entitled "Rats," the sobriquet being the nickname of the men engaged in the construction of the Rotherhithe Tunnel. The courage, coolness, and indifference to suffering of these "rats" could hardly be surpassed even by soldiers on the battlefield. "An East-End Heroine" contains a warning to journalists, although the moral is not pressed home unduly, and all the leaves of Col. Grant's "Diary" will be found well worth reading.

**The New Physiology in Surgical and General Practice.** By A. Rendle Short, M.D., B.S.C., F.R.C.S. Fourth edition. (Bristol: John Wright & Sons, Ltd.)

The previous edition of this extremely valuable and illuminating book was published just before the war, and it is unnecessary to explain what a vast amount of new information has come to hand during the intervening five years. Much of the book is, indeed, entirely new, but the main objects remain the same—namely, to sift from physiology that which is likely to be of value in the actual diagnosis and treatment of patients. The chapters on food deficiency diseases, the blood and spleen, surgical shock, the spinal cord, and the functions of the cortex have been rewritten almost in their entirety, and if we say that in its new form the author's publication has more than maintained its already high reputation, no greater praise is necessary.

**Diabetic Dietary and Cookery.** By P. J. Cammidge, M.D. (University of London Press: Hodder & Stoughton, Ltd. Pp. 222. Price 10s. 6d. net.)

Within the last few years many of the old dietary rules and, indeed, some of what were not so long ago regarded as fundamental principles in the treatment of diabetes, have been changed out of all recognition. In the volume before us Dr. Cammidge sets forth current views on the treatment of diabetes very fully and concisely. He rightly makes a great point of the need for enlisting the intelligent co-operation of the patient; and he is particularly strong on the practical aspect of the matter, such as cookery and recipes. The book is essentially one for the general practitioner's bookshelf of essential reference books, and can also be entrusted to patients who are of good education and well-balanced temperament.

